# Exercise as a Potential Intervention to Modulate Cancer Outcomes in Children and Adults?

**DOI:** 10.3389/fonc.2020.00196

**Published:** 2020-02-21

**Authors:** Sabine Kesting, Peter Weeber, Martin Schönfelder, Bernhard W. Renz, Henning Wackerhage, Irene von Luettichau

**Affiliations:** ^1^Kinderklinik München Schwabing, Department of Pediatrics and Children's Cancer Research Center, TUM School of Medicine, Technical University of Munich, Munich, Germany; ^2^Chair of Preventive Pediatrics, Department of Sport and Health Sciences, Technical University of Munich, Munich, Germany; ^3^Exercise Biology, Department of Sport and Health Sciences, Technical University of Munich, Munich, Germany; ^4^Department of General, Visceral, and Transplantation Surgery, Hospital of the University Munich, Munich, Germany; ^5^German Cancer Consortium (DKTK), Partner Site Munich; and German Cancer Research Center (DKFZ), Heidelberg, Germany

**Keywords:** cancer, childhood cancer, tumor, exercise training, molecular mechanisms, adjunct therapy

## Abstract

Exercise is recommended for the healthy population as it increases fitness and prevents diseases. Moreover, exercise is also applied as an adjunct therapy for patients with various chronic diseases including cancer. Childhood cancer is a rare, heterogeneous disease that differs from adult cancer. Improved therapeutic strategies have increased childhood cancer survival rates to above 80% in developed countries. Although this is higher than the average adult cancer survival rate of about 50%, therapy results often in substantial long-term side effects in childhood cancer survivors. Exercise in adult cancer patients has many beneficial effects and may slow down tumor progression and improve survival in some cancer types, suggesting that exercise may influence cancer cell behavior. In contrast to adults, there is not much data on general effects of exercise in children. Whilst it seems possible that exercise might delay cancer progression or improve survival in children as well, there is no reliable data yet to support this hypothesis. Depending on the type of cancer, animal studies of adult cancer types show that the exercise-induced increase of the catecholamines epinephrine and norepinephrine, have suppressive as well as promoting effects on cancer cells. The diverse effects of exercise in adult cancer patients require investigating whether these results can be achieved in children with cancer.

## Introduction and Background

Childhood cancer contributes about 1% to all malignant diseases worldwide (1) and the incidence rate of cancer in children aged 0–19 years in the USA from 1975 to 2016 varies between 14.7 and 19.3 cases per 100,000 per year with an increasing trend (2). Thanks to more effective therapies, the 5 year survival has increased to 84.8% in the USA in 2016 in children aged 0–19 years (2). The success in survival rates regarding childhood cancer has been achieved by a better understanding of the biology, improved diagnostics, stratification based on biomarkers as well as monitoring of disease and treatment (3) and by the high adherence rate to clinical studies (4). The drawback of the success rate are major long-term side effects (5). The International Guideline Harmonization Group for Late Effects of Childhood Cancer studies long-term effects to optimize the quality of care and to improve the quality of life for childhood cancer survivors (www.ighg.org^1^). Currently, around 37,000 survivors of childhood cancer are subjected to long-term follow-up programs in Germany (6) and more than 430,000 survivors of childhood cancer are living in the USA (7), representing 0.05% and 0.13% of the whole population, respectively. The incidence of childhood cancer is similar in Germany and the 15 year survival rate under the age of 15 has reached 82% (6). Worldwide, the incidence of childhood (0–14 years) cancer is 141 per million person years (8). This means that 215.000 children under the age of 15 and 80,000 adolescents between the age of 15 and 19 are globally diagnosed with cancer per year (8). Adult and childhood cancer not only differs regarding incidence and survival, but also with respect to biological features (9). This needs to be considered when transferring exercise oncology evidence from adults to children.

Physical activity and structured exercise programs not only help to prevent many chronic diseases, but exercise can also be an effective additive treatment strategy (10). This might also be true for cancer. An analysis of 1.44 million adults demonstrates an association between physical activity and reduced risk for at least 13 types of cancer (11). Exercise is a safe and feasible intervention and improves the psychosocial and physiological well-being of cancer patients. Moreover, observational studies suggest that exercise may slow down tumor progression and increase survival in specific types of cancer (12, 13).

Whilst the effects of exercise on adult cancer patients are increasingly characterized (12), far less is known about the effects of exercise in children with cancer (14–18). In this narrative review, we summarize the psychosocial and physiological effects of exercise in adults and childhood cancer patients. Eventually, we discuss potential mechanisms by which exercise may directly affect cancer cell behavior. Because there is hardly any research published on mechanisms by which exercise affects childhood cancer cells, we also point out known mechanisms that could potentially be exploited to target childhood cancer.

## Effects of Exercise Training in Cancer Patients

Exercise not only improves fitness and prevents disease but is also an effective treatment strategy for many chronic diseases (10). In their comprehensive review, the authors summarize the evidence for the prescription of exercise as therapy in 26 different diseases including psychiatric, neurological, metabolic and cardiovascular diseases, musculo-skeletal disorders and cancer. In recent years exercise training has been increasingly utilized in cancer patients and in a position statement the Clinical Oncology Society of Australia (19) now recommends that exercise should be embedded as part of standard practice in cancer care. We divide the effects of exercise in cancer patients before, during and after treatment in two categories:

**General Effects:** Exercise has widely been shown to influence quality of life as well as physical health. Examples for general effects of exercise training are an increase of cardiorespiratory fitness, increased muscular mass or a decreased cancer-induced fatigue (12).

**Direct Effects:** Exercise-regulated factors may directly affect features of cancer cells, such as self-sufficiency in growth signals, evading apoptosis or inducing angiogenesis, which are known as the hallmarks of cancer (20) or they may contribute to recruiting immune cells toward the tumor (21).

Research in exercise oncology has traditionally focused on the general effects of exercise in cancer patients. However, recent mechanistic and functional studies have reported direct effects of exercise on cancer cells (13, 22, 23). These direct effects will be described in chapter 4 “Direct effects of exercise on cancer cells” of this review.

The exercise oncology literature has recently been summarized in a systematic review of 679 exercise intervention studies of 50.112 mainly adult patients suffering from 25 different types of cancer (12). This review and other reviews on exercise and cancer (24–27) conclude that:

- Exercise training is a safe and feasible intervention in different types of cancer during and after cancer therapy.
- Exercise training can improve body composition, cardiovascular fitness, muscle strength, psychological well-being, health-related quality of life, and can reduce depression incidence, fatigue and helps to prevent or treat cancer cachexia (28, 29). Exercise can also positively influence long-term adverse events of treatment in cancer patients such as weight gain, metabolic dysfunction, endocrine disturbances, cardiotoxicity and the risk of developing cardiovascular diseases.

### General Effects of Exercise Training in Children and Adults With Cancer

Because childhood cancer is a rare and heterogeneous disease, there are few studies typically with fewer subjects, mixed cancer types and of a lower methodological quality on the effect of exercise in children with cancer when compared to studies in adult patients. Specifically, in the review of Christensen et al., only 32 out of 679 of the analyzed studies were conducted in children with cancer (12). Four reviews have summarized the findings on exercise in children with cancer based on just 30 studies covering all major childhood cancer types (14–17). Most of the studies were conducted in patients treated for acute lymphoblastic leukemia representing the most often diagnosed childhood cancer. The conclusions of these four reviews for exercise in children are described together with findings in adult cancer patients. By presenting current evidence existing in adult vs. childhood cancer patients together, we aim to facilitate the assessment of differences.

#### Safety and Feasibility

Similar to adults with cancer across the broad range of entities and phases of therapy (12), exercise is a safe and feasible intervention in children during acute inpatient care (17) and after cancer treatment (15). This conclusion is based on the finding that none of the included studies reported any adverse events (14, 16).

#### Physical Outcomes

A meta-analyses of 34 RCTs including 4,519 adult patients with cancer shows that exercise significantly improves physical fitness both during and after treatment (30). Different demographic or clinical characteristics did not affect the effectiveness of exercise on physical fitness. In children with cancer, exercise may positively influence the physical fitness, but mainly in patients with acute lymphoblastic leukemia, within an age range of 1–18 years (14, 15). Three out of five studies reported a significant increase of muscle strength after combined exercise training including mobility, resistance, and aerobic training in patients aged 4–16 years during treatment (16, 31–33). Similarly, cardiorespiratory fitness assessed for example through a 9 min walking test significantly increased in two out of five studies in patients with acute lymphoblastic leukemia, aged 5–12 years (16, 33, 34). There is a trend that a combined exercise training intervention including mobility, aerobic, and resistance training increases flexibility in joints and muscles in patients aged 1–17 years and during treatment of acute lymphoblastic leukemia, but the results are not significant (15, 16). Exercise has no clear effect on body composition. Hartman et al. reported a favorable reduction of the body mass index and body fat in the exercise vs. the control group in a 2 year supervised and home-based exercise program including mobility, endurance and functional training during treatment for acute lymphoblastic leukemia aged 1–17 years, but this was also not significant (35). Morales et al. (16) found that three other studies did not show any beneficial impact of a combined aerobic and resistance training on the body mass index or bone mineral density in patients aged 1–19 years treated for acute lymphoblastic leukemia. In summary, exercise can somewhat improve the physical fitness of children with cancer. Presumably, to achieve measurable training adaptations, exercise interventions must go beyond playful low intensity activities and include longer periods of moderate or vigorous intensity, which might be a challenge when dealing with children with cancer in a hospital.

#### Psychosocial Outcomes

Comprehensive reviews and meta-analyses have shown that exercise has small but significant effects on health-related quality of life, depression and fatigue in adult cancer patients (12, 30, 36). Mainly breast cancer patients were studied, but effects are also convincing in other types of cancer, such as prostate and hematological cancer (12). In contrast, there are no clear effects of exercise on psychosocial variables in children with cancer. Baumann et al. (14) concluded in their review regarding childhood cancer patients that there are positive effects of exercise on fatigue and health-related quality of life during treatment and survivorship. Braam et al. (15), who concluded that exercise does not significantly affect fatigue nor health-related quality of life in the reviewed randomized control trials in childhood cancer patients, contrast these data. Thus, at this point we need more experimental data to substantiate the evidence on this topic in children.

#### Mortality and Relapse

Physical activity is associated with improved survival (37) and reduced recurrence of breast (38), prostate (39) and colorectal cancer (40) in adults. In children, not enough cases have been studied to reliably judge whether exercise has an effect on survival or relapse. In a systematic review and meta-analysis of eight randomized control trials including 283 childhood cancer patients, the authors stated that exercise has neither positive nor negative effects on mortality or relapse risk (16).

In summary, exercise in children with cancer is understudied and there is an urgent need for high quality basic and translational research to exploit the effects of exercise in childhood cancer, thereby hopefully gaining better evidence on this topic. Currently, conclusions whether exercise in children with cancer has similar general effects as in adult cancer patients cannot be drawn. Moreover, it is important to identify joyous exercise programs specifically for in-patients that deliver a sufficiently high dose of resistance and endurance exercise for biological adaptions in children. These programs need to be playful and motivating using e.g., balls, obstacles, and suitable equipment for smaller children as well as appropriate tools for adolescents, e.g., dumb bells, cable pull, and ergometers for short bouts of high intensity training. The differentiation and age-appropriate adaption of exercise programs between children and adolescents might be beneficial. The group of adolescents and young adults (so called AYAs) is still understudied and robust evidence regarding specific and health-beneficial exercise programs is lacking (41). In adults, exercise seems to have a dose-dependent effect on cancer-specific mortality; however, the optimal dose of exercise is not known for adults nor for children (42). A special focus of future childhood exercise oncology studies should be to investigate whether exercise can be employed to reduce the often life-long consequences that are caused by highly toxic or mutilating treatments such as chemotherapy, surgery and radiotherapy delivered to a growing organism (4). Comprehensive and confirmatory long-term studies with childhood cancer patients subjected to exercise programs already during treatment are still missing due to the short period of implementation. Based on these investigations, clear training recommendations regarding the different types of exercise to target specific toxicities are required. The first promising results of an exploratory analysis suggest a mitigation of cardiovascular diseases and an improvement of bone mineral density in childhood cancer survivors due to adequate levels of physical activity (43).

## Direct Effects of Exercise on Cancer Cells

Much of the early research in exercise oncology has focused on the general effects of exercise in cancer patients. In contrast, recent basic research studies aim to identify molecular and cellular mechanisms by which physical activity or exercise training might directly change cancer cell behavior. These studies all investigate adult cancer types.

### Animal Models

Observational studies in animal models suggest that exercise has direct effects on cancer cells. Generally, the exercising muscles and other organs will alter the molecular and cellular composition of blood and thereby potentially influence tumor cells. Additionally, effects of exercise may be directly mediated by the nervous system, a part of the tumor microenvironment gaining increasing attention in cancer research (44–48). A recent systematic review analyzed mechanisms of aerobic exercise on cancer initiation, progression, and metastasis in animal models (49). They included a total of 53 studies and reported a considerable methodological heterogeneity, which impedes reliable comparison. Most of the reviewed studies worked with voluntary running, forced running or swimming. The majority of studies analyzed incidence and growth and found that exercise inhibits tumor initiation as well as growth in most instances. Three studies used models where metastases arose from primary tumors, that reported non-significant tumor inhibition in two cases (50, 51) and in one case accelerated tumor growth (52) with exercise. The authors conclude that the variety of outcomes is a result of poor methodological consistency and therefore they provide methodological and data reporting standards for preclinical studies in exercise oncology.

### Human Studies

**Figure 1:**
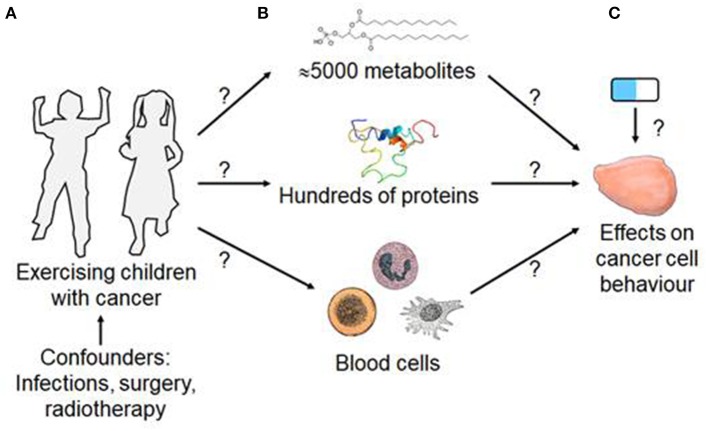
Conceptual framework of exercise-induced, blood-mediated effect on cancer cell behavior. **(A)** Structured exercise training of a child will affect the concentration of **(B)** blood components such as metabolites, proteins and blood cells. **(C)** Whilst blood changes will not correct the driver mutations that cause cancer, there is evidence that exercise-conditioned blood can affect the behaviors or hallmarks of cancer cells and may alter the responsiveness of cancer cells to anti-cancer drugs. [Blood cell images are from Gray's anatomy and in the public domain. The IGF-1 protein structure, shown above the “Hundreds of proteins” is from https://commons.wikimedia.org/w/index.php?curid=8820088. All other figures are drawn by HW].

In humans, exercise not only increases the concentration of many metabolites e.g., lactate (53), proteins (54), packed proteins in extracellular vesicles (55), or hormones such as catecholamines (56, 57). Exercise also affects blood cell numbers, especially those of immune cells, possibly altering immune responses in relation to time and intensity of exercise (58, 59). However, there is limited data on the effects of exercise on the immune system in children with cancer (14). One study demonstrated a significant increase of natural killer cell cytotoxicity in children with mixed tumor entities undergoing an allogeneic hematopoietic stem cell transplantation subjected to a 10 week exercise program with 60 min of combined aerobic and strength training with moderate-to-vigorous intensity (60). Another study only found a trend toward an interaction effect for natural killer cells expressing the immunoglobulin-like receptor KIR2DS4 in children with solid tumors during chemotherapy treatment conducting an exercise program including aerobic and strength training of at least 60 min with moderate-to-vigorous intensity for 20 weeks on average (31). Finally, exercise affects systemic and organ blood flow and body core temperature (61). This conceptual framework for the direct effects of exercise on cancer is illustrated in Figure 1.

The molecular mechanisms by which exercise can affect adult cancer cell behavior have been discussed extensively in several recent reviews. Direct cancer effects in adult patients include influence on proliferation, signal transduction, cancer metabolism, inflammation, and cancer-immune system interactions (13, 22, 23). Here, we describe an example of direct cancer effects of exercise with potential importance for childhood cancer.

## Catecholamine-Mediated Direct Effects of Exercise on Cancer Cells

### Human Studies

The exercise-induced stress hormones epinephrine (British English: adrenaline) and norepinephrine (British English: noradrenaline) not only regulate many acute adaptations to exercise but can also affect cancer cell signaling and behavior. The stimulation of catecholamines by exercise was first described in the early 1950s (56). Generally, the blood concentrations of epinephrine and norepinephrine increase with the intensity and duration of exercise (57), in both children and adolescents (62). There are no major differences in the catecholamine response to exercise in children and adults (63). Epinephrine and norepinephrine bind to α1,2, or β1,2,3 adrenergic receptor isoforms (64). Notably, many cancer cells express adrenergic receptor isoforms. Catecholamines binding to their receptors can potentially modulate inflammation, angiogenesis, tissue invasion, epithelial-to-mesenchymal transformation and affect cellular immune responses in cancer cells (64). Evidence that catecholamine signaling is important in cancer originates in epidemiological studies that report an association between β-blocker medication and reduced tumor progression (64). In children, β-blockers are used as a first-line treatment for infantile hemangioma (65).

### Animal Models and Cell Culture Studies

In addition to humans, Pedersen et al. demonstrated that voluntary running of mice before but not after injection of B16, melanoma cells reduced the growth and volume of the developing tumors. Moreover, there were more natural killer cells in the xenotransplant tumors of the mice that had exercised. Further experiments demonstrated that this was dependent on catecholamines as receptor blockade with the unselective β-blocker propranolol prevented this effect (21). In a further cell culture based study, the Hojman group published data suggesting that catecholamines can inhibit Hippo effector YAP (yes-associated protein) in breast cancer cells, a known regulator of proliferation (66, 67). The Copenhagen team who provided evidence that catecholamines may mediate some of the direct exercise effects in breast cancer followed this up (68). Together, this data suggests that exercise-induced modulation of catecholamines can directly inhibit cancer cell seeding and growth.

In contrast, Renz et al., who found that chronic restraint stress—not exercise-induced—, promoted the release of catecholamines in *KRAS*-mutant mice thereby significantly increasing incidence of pancreatic adenocarcinomas. Moreover, they also reported observational data that pancreatic adenocarcinoma patients treated with unselective beta-blockers for other reasons survived significantly longer than patients that did not receive this specific medication (46). These data suggest that catecholamines may have adverse effects on a subgroup of malignant tumors or at least in different circumstances. This finding is in line with other basic and clinical studies that report an association between β-blocker medication and better survival in ovarian cancer in mouse models (69, 70).

In summary, current evidence suggests that catecholamines can have both positive and negative effects on cancer cells presumably depending on circumstance as well as on the type of cancer. The fact that exercise reduces cancer risk, may reduce its progression and improve survival in humans (11–13) suggests that either catecholamines inhibit cancer progression in most cases or that tumor promoting effects of catecholamines are compensated by other exercise-induced factors. For example, in a B16F10 melanoma xenotranplant mouse the tumors grow significantly more in the exercising than in the control mice (71). A key research focus in childhood cancer should therefore be to study the effect of catecholamines on childhood tumors. Furthermore, mechanistic research should divide the group of adults according to their age, since exercise seems to have different effects in different age-groups (57).

## Concluding Remarks

This narrative review summarizes the effects of exercise as an adjunct therapy in cancer patients. In adult cancer patients, exercise is an effective intervention to trigger many general, indirect effects that improve the condition and physical performance of the patient. However, this evidence is mainly based on studies with breast cancer patients. Therefore, further investigations are needed for other cancer types and the optimal dose of exercise. Moreover, there is increasing evidence that exercise also directly affects cancer cells. In children, these issues have not been sufficiently addressed.

Recent publications have shown that exercise can improve patient outcome in many different ways. The idea of using exercise to put a cancer patient into a “sweet spot” where drug effectiveness may be increased, toxicity reduced and life quality maintained or improved has not been explored extensively so far and therefore may be worth testing.

Consequently, the following issues should be a focus for researchers working in the field of childhood exercise oncology to gain knowledge especially regarding mechanisms by which exercise may influence childhood cancer cell lines.

## Future Research Needs

- Based on the finding that the general effects of exercise in children with cancer are small, a focus of future research should be to design and evaluate specific, joyous exercise interventions that allow delivering a higher dose of moderate-to-vigorous exercise to trigger clinically meaningful general adaptations in children with cancer.
- Future studies should also seek to determine whether exercise interventions can be used to protect children from the negative long-term consequences that result from treating a growing organism with aggressive therapies.
- Large, multi-center high quality studies should be performed to confirm whether exercise slows cancer progression and improves survival in children as has been shown in some adult tumors (12, 13).
- As reliable epidemiological data on exercise and childhood cancer will take a long time due to the low case numbers, one strategy could be to use childhood cancer cell lines or childhood patient-derived murine xenotransplant models to study how exercise, exercise-conditioned sera or specific exercise-induced blood factors alter the behavior of childhood cancer cells.
- Research on childhood cancer and exercise may even play an exemplary role in some respects regarding cancer specific processes. Due to a less complex mutational landscape and a less immunogenic character of childhood cancer entities compared to adult cancer, especially the influence of exercise on the immune system should be investigated further to reveal novel insights.
- Some tumors (e.g., cerebellar tumors) already originate during early embryonal development (72, 73). Therefore, in children, cancer prevention through lifestyle changes should not be the main focus. However, considering the emerging evidence it could be assumed that exercise induced manipulation of tumor cells by influencing the metabolome and proteome could potentially be implemented to reduce relapse, mitigate late effects, and facilitate therapy.

## Author Contributions

HW and IL developed the concept of the review and were the main writers of the manuscript. SK and PW contributed equally to the concept, collected and analyzed data, and contributed to the writing. MS and BR contributed to the concept and revised the manuscript. All authors read and approved the final manuscript.

### Conflict of Interest

The authors declare that the research was conducted in the absence of any commercial or financial relationships that could be construed as a potential conflict of interest.

## Footnotes

^1^*International Guideline Harmonization Group for Late Effects of Childhood Cancer*. Available online at: www.ighg.org (accessed October 16, 2019).
